# In Vitro Inhibition of Growth, Biofilm Formation, and Persisters of *Staphylococcus aureus* by Pinaverium Bromide

**DOI:** 10.1021/acsomega.3c00340

**Published:** 2023-03-06

**Authors:** Ting Mao, Bao Chai, Yanpeng Xiong, Hongyan Wang, Lei Nie, Renhai Peng, Peiyu Li, Zhijian Yu, Fang Fang, Xianqiong Gong

**Affiliations:** † Hepatology Center, Xiamen Hospital, Beijing University of Chinese Medicine, Xiamen 361001, China; ‡ Department of Dermatology, Shenzhen Nanshan People’s Hospital and the 6th Affiliated Hospital of Shenzhen University Medical School, Shenzhen 518052, China; § Department of Infectious Diseases and Shenzhen Key Lab of Endogenous Infection, Shenzhen Nanshan People’s Hospital and the 6th Affiliated Hospital of Shenzhen University Medical School, Shenzhen 518052, China; ∥ Department of Infectious Diseases and Department of General Medicine, the Key Lab of Endogenous Infection, Shenzhen Nanshan People’s Hospital and the 6th Affiliated Hospital of Shenzhen University Medical School, Shenzhen 518052, China

## Abstract

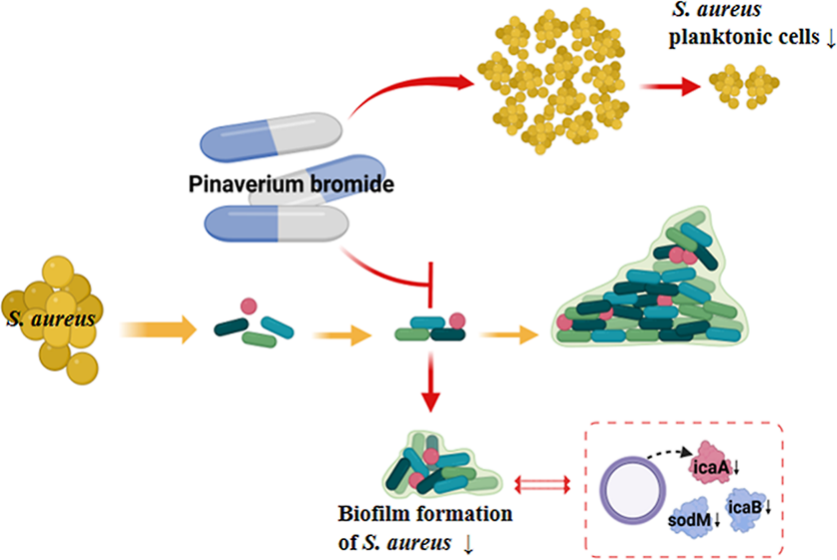

Biofilm or persister cells formed by *Staphylococcus aureus* are closely related to pathogenicity. However, no antimicrobials exist to inhibit biofilm formation or persister cells induced by *S. aureus* in clinical practice. This study found that pinaverium bromide had antibacterial activity against *S. aureus*, with the MIC_50_/MIC_90_ at 12.5/25 μM, respectively. Pinaverium bromide (at 4 × MIC) showed a rapid bactericidal effect on *S. aureus* planktonic cells, and it was more effective (at least 1-log_10_ cfu/mL) than linezolid, vancomycin, and ampicillin at 4 h of the time-killing test. Pinaverium bromide (at 10 × MIC) significantly inhibited the formation of *S. aureus* persister cells (at least 3-log_10_ cfu/mL) than linezolid, vancomycin, and ampicillin at 24, 48, 72, 96, and 120 h of the time-killing test. Biofilm formation and adherent cells of *S. aureus* isolates were significantly inhibited by pinaverium bromide (at 1/2 or 1/4 × MICs). The fluorescence intensity of the membrane polarity of *S. aureus* increased with the treatment of pinaverium bromide (≥1 × MIC), and the MICs of pinaverium bromide increased by 4 times with the addition of cell membrane phospholipids, phosphatidyl glycerol and cardiolipin. The cell viabilities of human hepatocellular carcinoma cells HepG2 and Huh7, mouse monocyte-macrophage cells J774, and human hepatic stellate cells LX-2 were slightly inhibited by pinaverium bromide (<50 μM). There were 54 different abundance proteins detected in the pinaverium bromide-treated *S. aureus* isolate by proteomics analysis, of which 33 proteins increased, whereas 21 proteins decreased. The abundance of superoxide dismutase sodM and ica locus proteins icaA and icaB decreased. While the abundance of global transcriptional regulator spxA and Gamma-hemolysin component B increased. In conclusion, pinaverium bromide had an antibacterial effect on *S. aureus* and significantly inhibited the formation of biofilm and persister cells of *S. aureus*.

## Introduction

*Staphylococcus aureus* is one of the leading causes of hospital- and community-acquired infections worldwide, which causes a wide variety of infectious diseases from common skin infections such as folliculitis to deep and fatal infections such as pneumonia, endocarditis, and so forth.^1^ Due to the extensive use of antimicrobials, drug-resistant *S. aureus* infection, especially the methicillin-resistant *S. aureus* (MRSA), has caused serious clinical and public health problems and attracted more and more attention.^2^ What is more serious is that, in recent years, the widespread emergence of vancomycin intermediate-resistant *S. aureus* (VISA/hVISA) and linezolid-resistant strains has brought greater challenges and difficulties to the clinical treatment of *S. aureus* infections.^3,4^ Therefore, it is still necessary to search for new antimicrobials against *S. aureus* infections.

At present, in addition to drug resistance, *S. aureus* can also form biofilms and persisters on the surface of human tissues and organs or implants, causing chronic infections and non-healing.^5^ The biofilm formed by *S. aureus* is composed of proteins, extracellular polysaccharides (EPS), and some small molecules (such as eDNA), which are conducive to their survival in extreme environments and can greatly limit the diffusion and penetration of commonly used antimicrobials, making them difficult to be completely cured.^6^ Now, it was found that *S. aureus* can produce persisters under the influence of antimicrobials, and *S. aureus* biofilms also contain a large number of persisters, which are resistant to antimicrobials through complex dormancy mechanisms, resulting in repeated infections and difficulty to treat.^5,7^ Thus, now, it remains critical to explore new antimicrobials with an inhibitory effect on the biofilm formation and persisters of *S. aureus*.^8^ However, the development of new antimicrobials often requires a long time, high investment costs, and huge risks.^9^ Interestingly, finding new drugs with antibacterial effect from old drugs has become a cost-effective method in recent years.^10^ Thus, this study aims to explore chemicals from the US Food and Drug Administration (FDA) approval drug library to inhibit the growth, biofilm formation, and persisters of *S. aureus*.

## Results

### Pinaverium Bromide Has Antibacterial Activity on *S. aureus*

To explore chemicals inhibiting growth and biofilm formation of *S. aureus*, the FDA-Approved Drugs Library was used to screen by crystal violet staining. The clinical *S. aureus* CHS101 isolate was used as the screening strain.^11^*S. aureus* CHS101 isolate (with chemicals at 50 μM) was inoculated into 96 polystyrene microtiter plates with tryptic soy broth with 0.5% glucose (TSBG). After 24 h of static incubation, the growth of planktonic cells in the culture supernatant and biofilms formed at the bottom of microtiter plates were measured. The present study found that the growth of planktonic cells and biofilm formation of *S. aureus* CHS101 isolate were significantly inhibited by pinaverium bromide (50 μM). Thus, the minimum inhibitory concentrations (MICs) and minimum bactericidal concentrations (MBCs) of pinaverium bromide against 56 *S. aureus* and 5 *Enterococcus faecalis* isolates were determined by the broth macrodilution method. As shown in Tables 1 and S1, the MICs of pinaverium bromide against 56 *S. aureus* isolates ranged from 6.25 to 50 μM and with the MIC_50_/MIC_90_ at 12.5/25 μM, respectively. Pinaverium bromide also indicated antibacterial effect on *E. faecalis*, and both with the MIC_50_/MIC_90_ at 50/50 μM against five *E. faecalis* isolates.

**Table 1:** Antimicrobial Susceptibilities of Pinaverium Bromide against *S. aureus* and *E. faecalis*^a^

	the MICs (μM) of pinaverium bromide					
bacterial species	6.25	12.5	25	50	100	MIC_50_/MIC_90_
**MSSA(*n*** = **40)**	1	30	6	3		12.5/25
**MRSA(*n*** = **16)**		13	1	1	1	12.5/12.5
E.faecalis **(*n*** = **5)**			2	3		50/50

a Note: MIC, minimum inhibitory concentration; MSSA, methicillin-sensitive *S. aureus*; MRSA, methicillin-resistant *S. aureus*; and MIC_50_/MIC_90_, the minimum inhibitory concentration required for 50 or 90% of bacterial growth inhibition.

### Rapid Bactericidal Effect of Pinaverium Bromide on *S. aureus* Planktonic Cells

**Figure 1:**
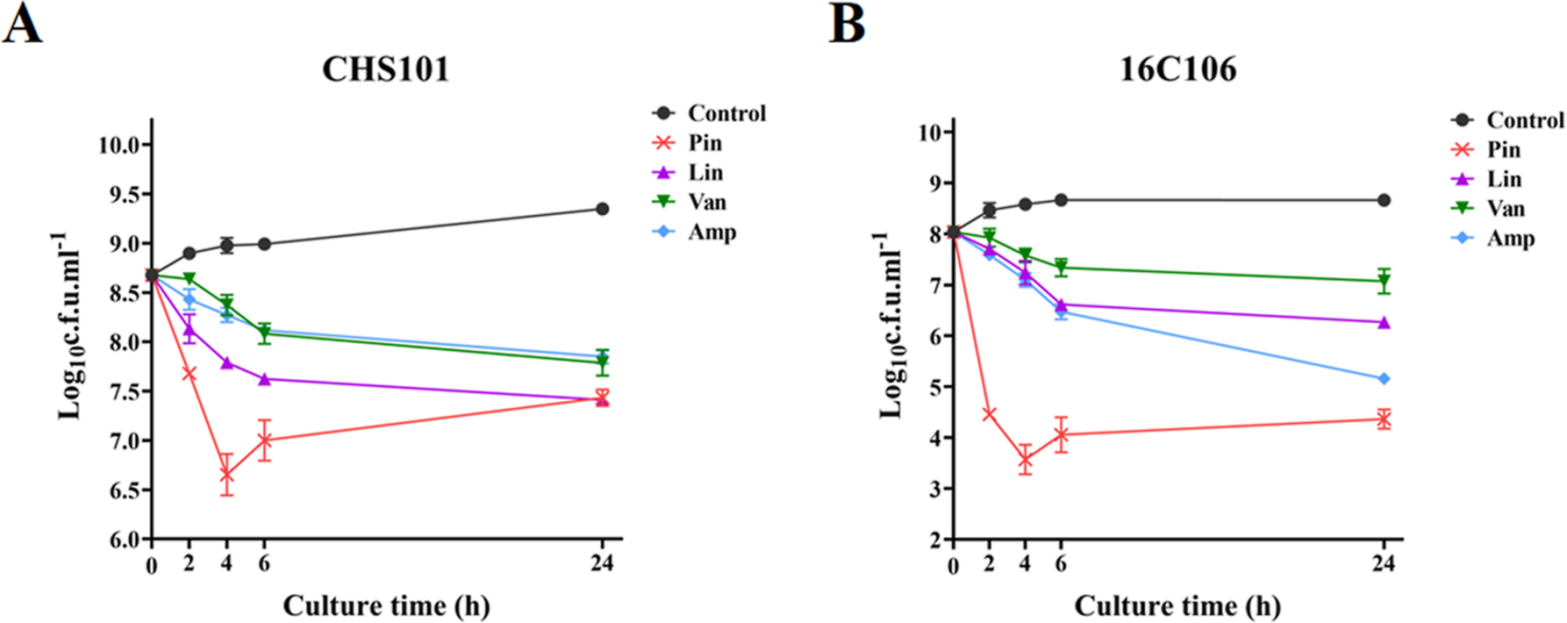
Antibacterial effect of pinaverium bromide on the planktonic cells of *S. aureus* and *E. faecalis*. The *S. aureus* CHS101 isolate (A) and *E. faecalis* 16C106 isolate (B) during the logarithmic growth phase were treated with pinaverium bromide for 24 h. The planktonic cells were determined by cfu numbers. The data presented were the average of three independent experiments (mean ± SD). Pin, pinaverium bromide; Lin, linezolid; Van, vancomycin; and Amp, ampicillin. All antimicrobials were used at 4 × MIC.

The rapid bactericidal effect of pinaverium bromide on *S. aureus* planktonic cells were determined by the time-killing test and compared with that of linezolid, vancomycin, and ampicillin (all used at 4 × MIC).^12^ Pinaverium bromide showed a rapid bactericidal effect on *S. aureus* CHS101 planktonic cells and killed more planktonic cells (at least 1-log_10_ cfu/mL) than linezolid, vancomycin, and ampicillin at the 4 h of the time-killing test (Figure 1). The similar results were also observed in the *E. faecalis* 16C106 isolate.

### Production of *S. aureus* Persister Cells Were Significantly Inhibited by Pinaverium Bromide

**Figure 2:**
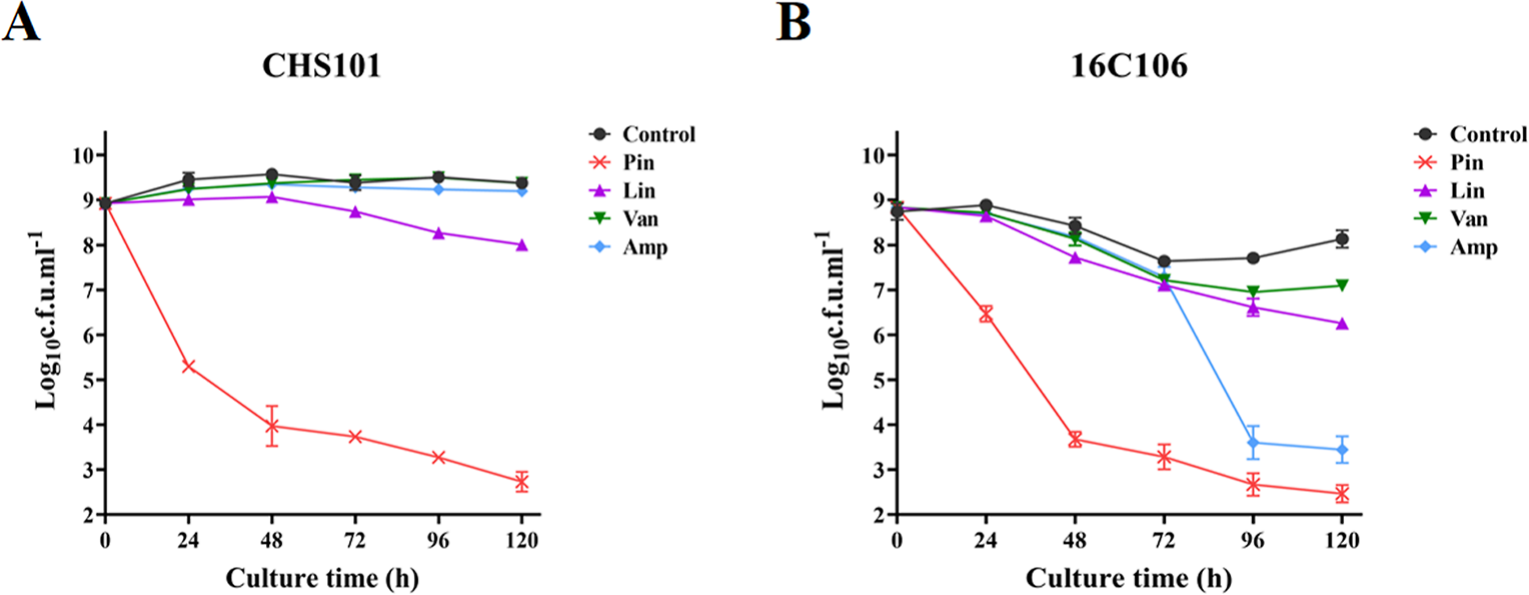
Inhibitory effect of pinaverium bromide on the formation of *S. aureus* and *E. faecalis* persister cells. The *S. aureus* CHS101 isolate (A) and *E. faecalis* 16C106 isolate (B) during stationary growth phase were treated with pinaverium bromide for 120 h. The planktonic cells were determined by cfu numbers. The data presented were the average of three independent experiments (mean ± SD). Pin, pinaverium bromide; Lin, linezolid; Van, vancomycin; and Amp, ampicillin. All antimicrobials were used at 10 × MIC.

Now it is also found that persister cells played an important role in antimicrobials resistance and biofilm formation of bacteria.^8,13^ Thus, the present study investigated the inhibitory effect of pinaverium bromide on the production of *S. aureus* persister cells at high concentration and compared with that of linezolid, vancomycin, and ampicillin (all used at 10 × MIC).^12^ Pinaverium bromide significantly inhibited the production of *S. aureus* CHS101 persister cells (at least 3-log_10_ cfu/mL) than linezolid, vancomycin, and ampicillin at the 24, 48, 72, 96, and 120 h of the time-killing test (Figure 2). The similar results were also observed in the *E. faecalis* 16C106 isolate at the 48 and 72 h of the time-killing test.

### Biofilm Formation of *S. aureus* Was Inhibited by Subinhibitory Concentrations of Pinaverium Bromide

**Figure 3:**
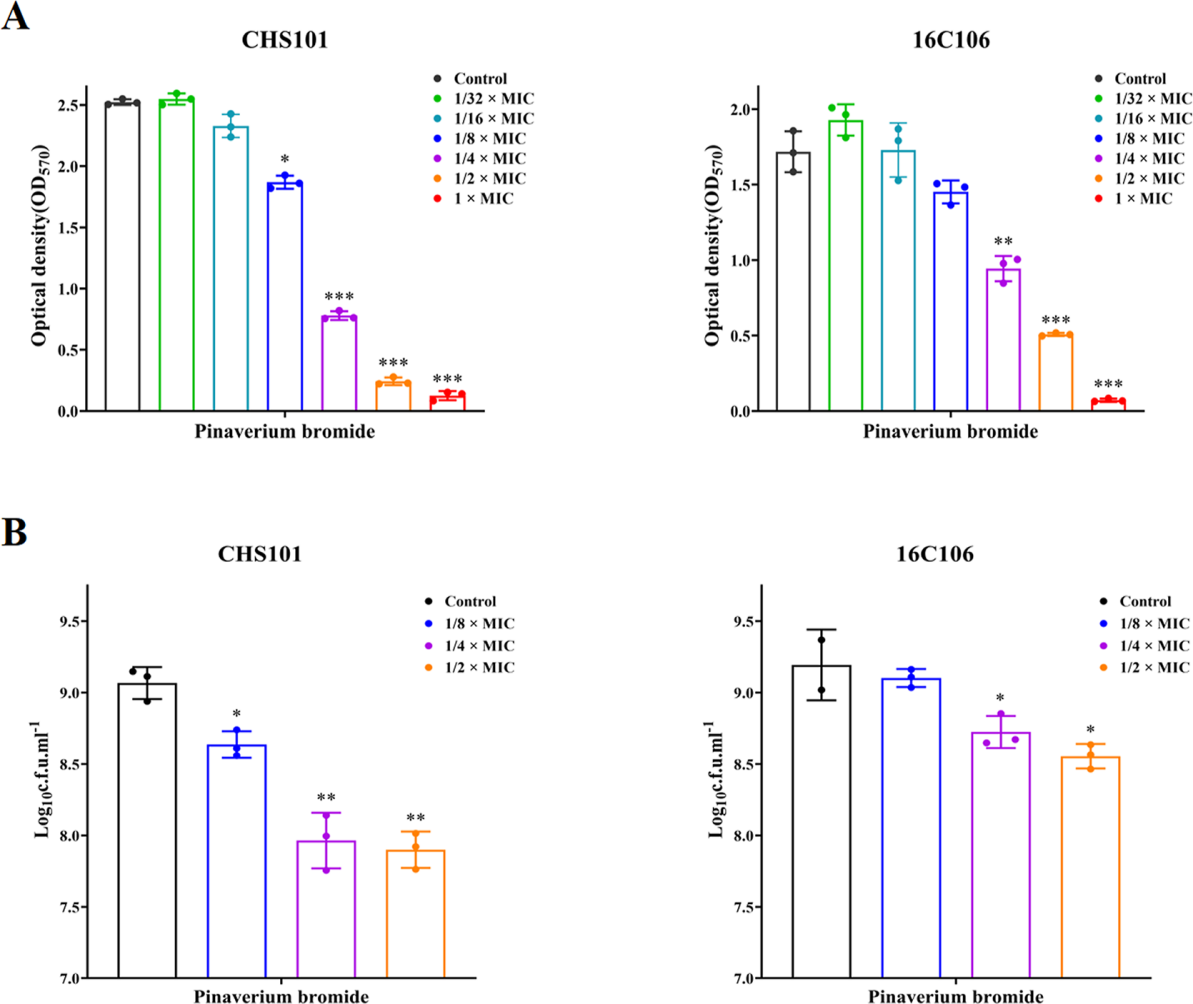
Effect of sub-MIC concentrations of pinaverium bromide on the biofilm formation and adherent cells of *S. aureus* and *E. faecalis*. (A) The *S. aureus* CHS101 isolate and *E. faecalis* 16C106 isolate were treated with pinaverium bromide for 24 h, and the biofilm biomasses were determined by crystal violet staining. (B) The *S. aureus* CHS101 isolate and *E. faecalis* 16C106 isolate were treated with pinaverium bromide for 24 h, the adherent cells in biofilms were collected and determined by cfu numbers. The data presented were the average of three independent experiments (mean ± SD). Compared with control, *: *P* < 0.05; **: *P* < 0.01; and ***: *P* < 0.001 (Student’s *t*-test). MIC, minimum inhibitory concentration.

**Figure 4:**
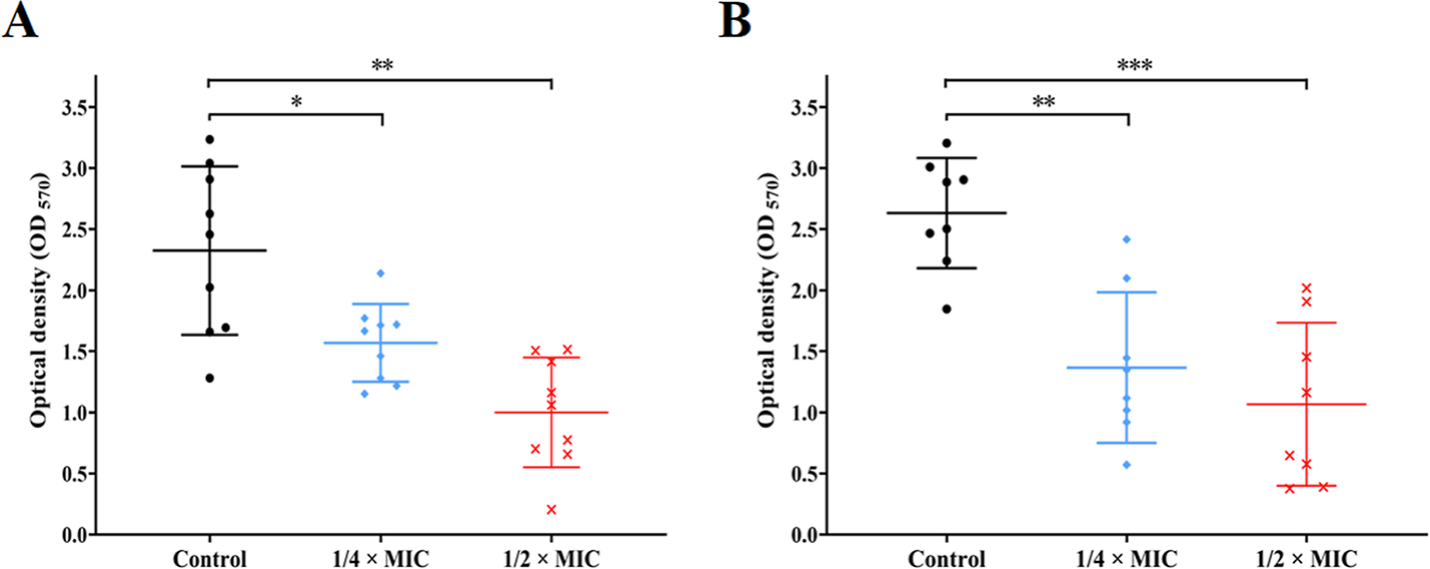
Effect of 1/4 or 1/2 × MICs of pinaverium bromide on the biofilm formation of *S. aureus* isolates. The nine MSSA (A) and eight MRSA (B) were treated with pinaverium bromide at 1/4 or 1/2 × MICs for 24 h, and the biofilm biomasses were determined by crystal violet staining. The data presented were the results of three independent experiments and take the mean value of three independent experiments for each strain for analysis. The results of *S. aureus* isolates in each group were presented as mean ± 95% confidence interval. Compared with control, *: *P* < 0.05; **: *P* < 0.01; and ***: *P* < 0.001 (Student’s *t*-test). MIC, minimum inhibitory concentration; MSSA, methicillin-sensitive *S. aureus*; and MRSA, methicillin-resistant *S. aureus*.

Interestingly, this study found that biofilm formation and adherent cells of *S. aureus* CHS101 and *E. faecalis* 16C106 isolates were significantly inhibited by 1/2 × or 1/4 × MICs of pinaverium bromide (Figure 3). This interesting finding was also confirmed in more *S. aureus* clinical isolates (Figure 4). However, the present study demonstrated that different concentrations of pinaverium bromide (from 1/4 × to 8 × MICs) had no eradicating effect on the established biofilms of *S. aureus* CHS101 and *E. faecalis* 16C106 isolates (Figure S1).

### Disruption of the Membrane Polarity of *S. aureus* by Pinaverium Bromide

**Figure 5:**
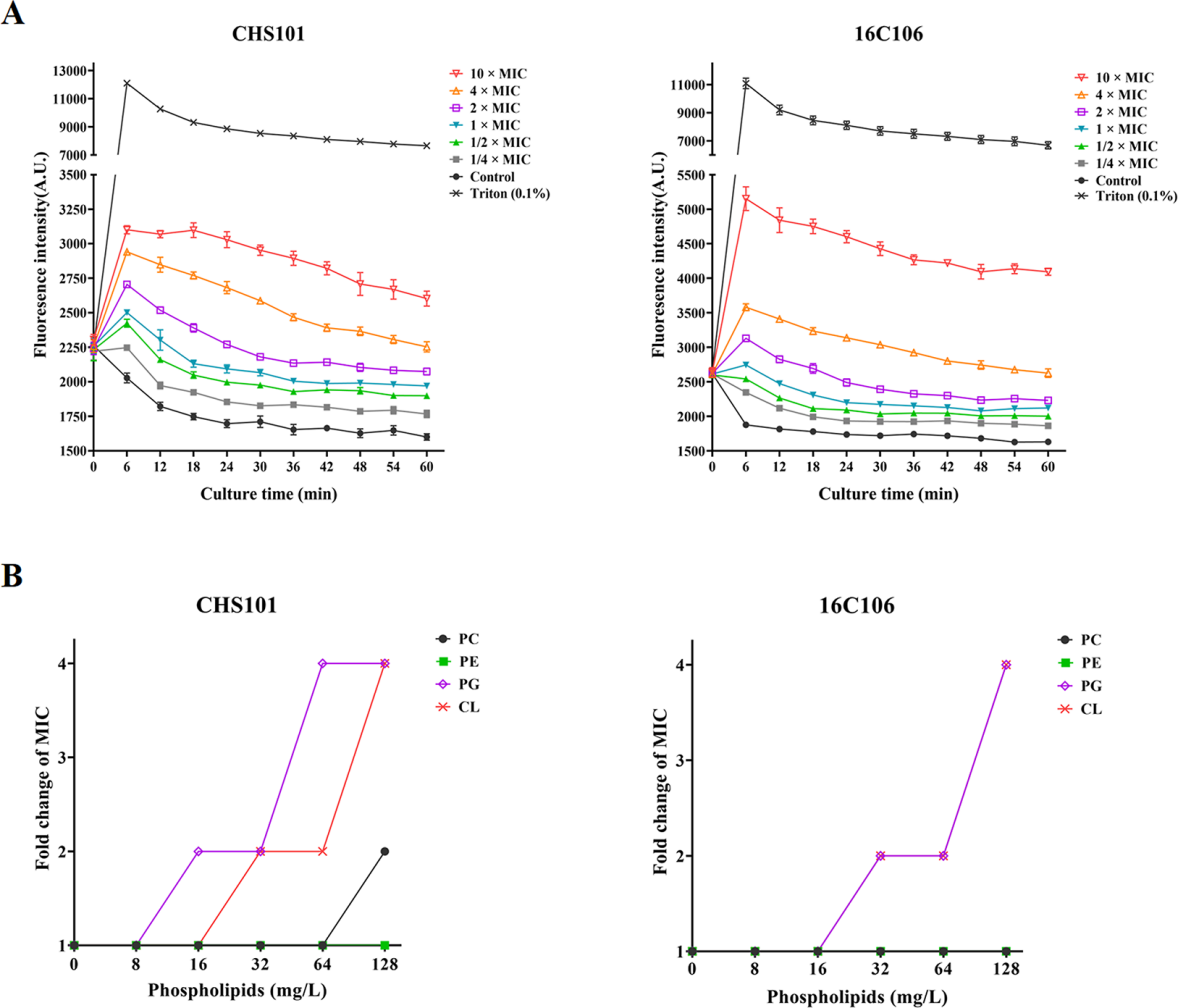
Effect of pinaverium bromide on the cell membrane of *S. aureus* and *E. faecalis*. (A) The *S. aureus* CHS101 isolate and *E. faecalis* 16C106 isolate were stained with fluorescent dye DIBAC4(3), then treated with different concentrations of pinaverium bromide, and finally, the fluorescence intensity was detected to evaluate the membrane polarity of *S. aureus* and *E. faecalis*. (B) The MICs of pinaverium bromide against the *S. aureus* CHS101 isolate and *E. faecalis* 16C106 isolate (including four additional membrane phospholipids) were determined to explore the role of membrane phospholipids in the antibacterial activities of pinaverium bromide. The data presented were the average of three independent experiments (mean ± SD). MIC, minimum inhibitory concentration; PC, phosphatidyl choline; PE, phosphatidyl ethanolamine; PG, phosphatidyl glycerol; and CL, cardiolipin.

To explore the impact of pinaverium bromide on the membrane polarity of *S. aureus*, DiBaC4(3) uptake assay was performed, and the results indicated that the fluorescence intensity was increased in the pinaverium bromide-treated (≥1 × MIC) *S. aureus* and *E. faecalis* (Figure 5A). In order to further investigate the role of cell membrane in the antibacterial activity of pinaverium bromide, the effect of four cell membrane phospholipids (phosphatidyl choline, phosphatidyl ethanolamine, phosphatidyl glycerol, and cardiolipin) on the MICs of pinaverium bromide was evaluated. As indicated in Figure 5B, the addition of phosphatidyl glycerol and cardiolipin can increase the MICs of pinaverium bromide against *S. aureus* and *E. faecalis* by 4 times.

### Cytotoxicity of Pinaverium Bromide

**Figure 6:**
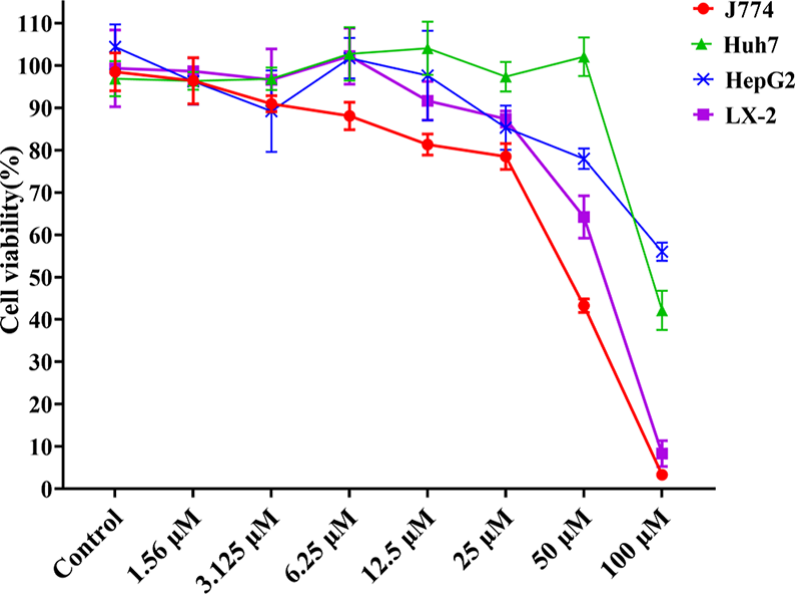
Effect of different concentrations of pinaverium bromide on the cell viabilities. Human hepatocellular carcinoma cells HepG2 and Huh7, mouse monocyte-macrophage cell J774, and human hepatic stellate cell LX-2 were treated with pinaverium bromide at different concentrations for 24 h. Cell viabilities were determined by the Cell Counting Kit-8 kit. The data presented were the average of three independent experiments (mean ± SD).

In order to explore the cytotoxicity of pinaverium bromide, the human hepatocellular carcinoma cells HepG2 and Huh7, mouse monocyte-macrophage cell J774, and human hepatic stellate cell LX-2 were selected, and cell viabilities were determined by the Cell Counting Kit-8 kit. As Figure 6 indicated, cell viabilities were significantly inhibited by the high concentrations of pinaverium bromide (at 50 or 100 μM).

### Proteomic Analysis of Different Abundance Proteins in *S. aureus* Isolate Treated with Pinaverium Bromide

**Figure 7:**
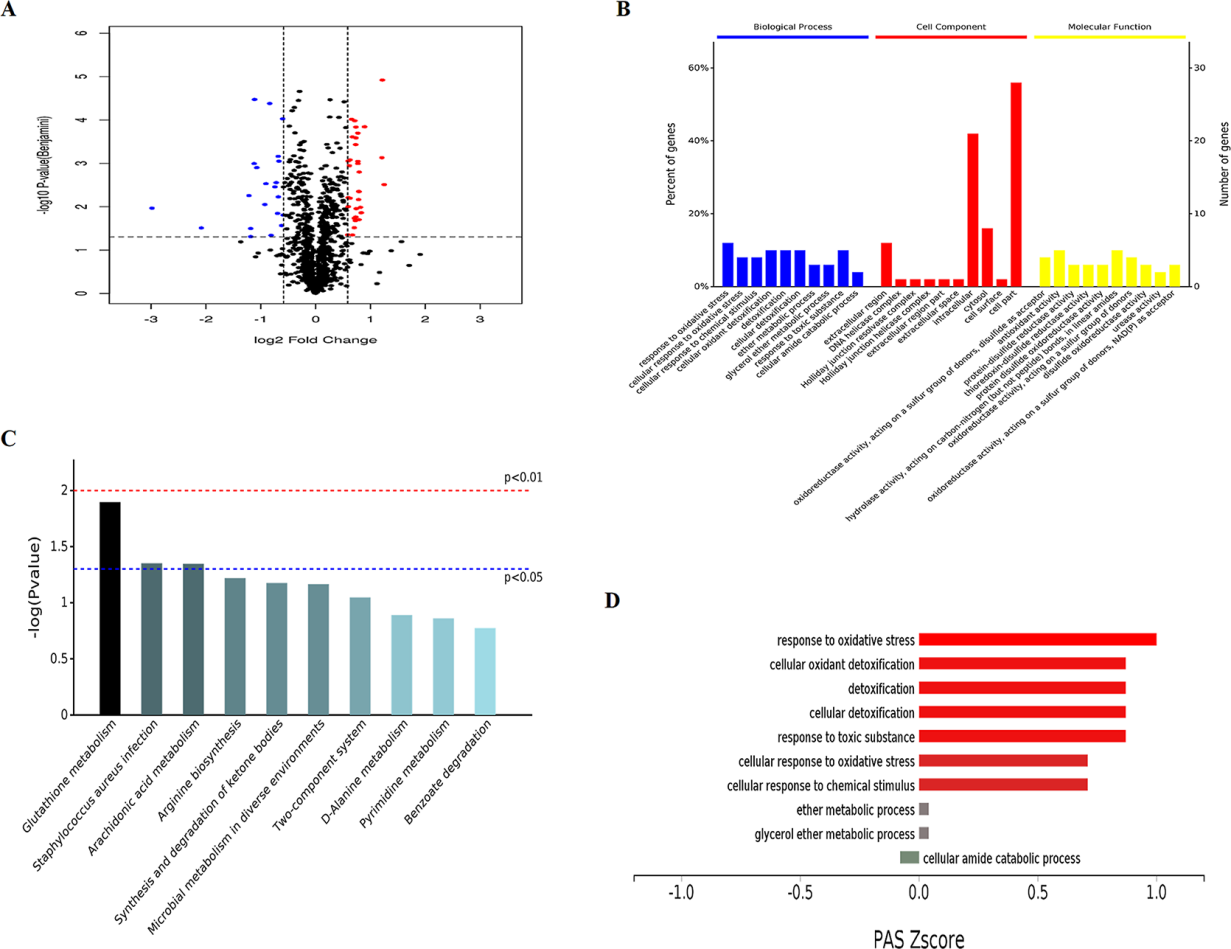
Different abundance proteins in pinaverium bromide-treated *S. aureus* isolate. (A) The different abundance proteins were shown in a volcano plot. The horizontal dotted line represents *P* ≤ 1.00 × 10^–2^. The vertical dotted line represents fold-change ≥1.5 or ≤0.667. (B) The molecular functions of different abundance proteins were classified by GO analysis. (C) The pathways of different abundance proteins were classified by KEGG analysis. (D) The PAS value of enriched biological processes.

**Figure 8:**
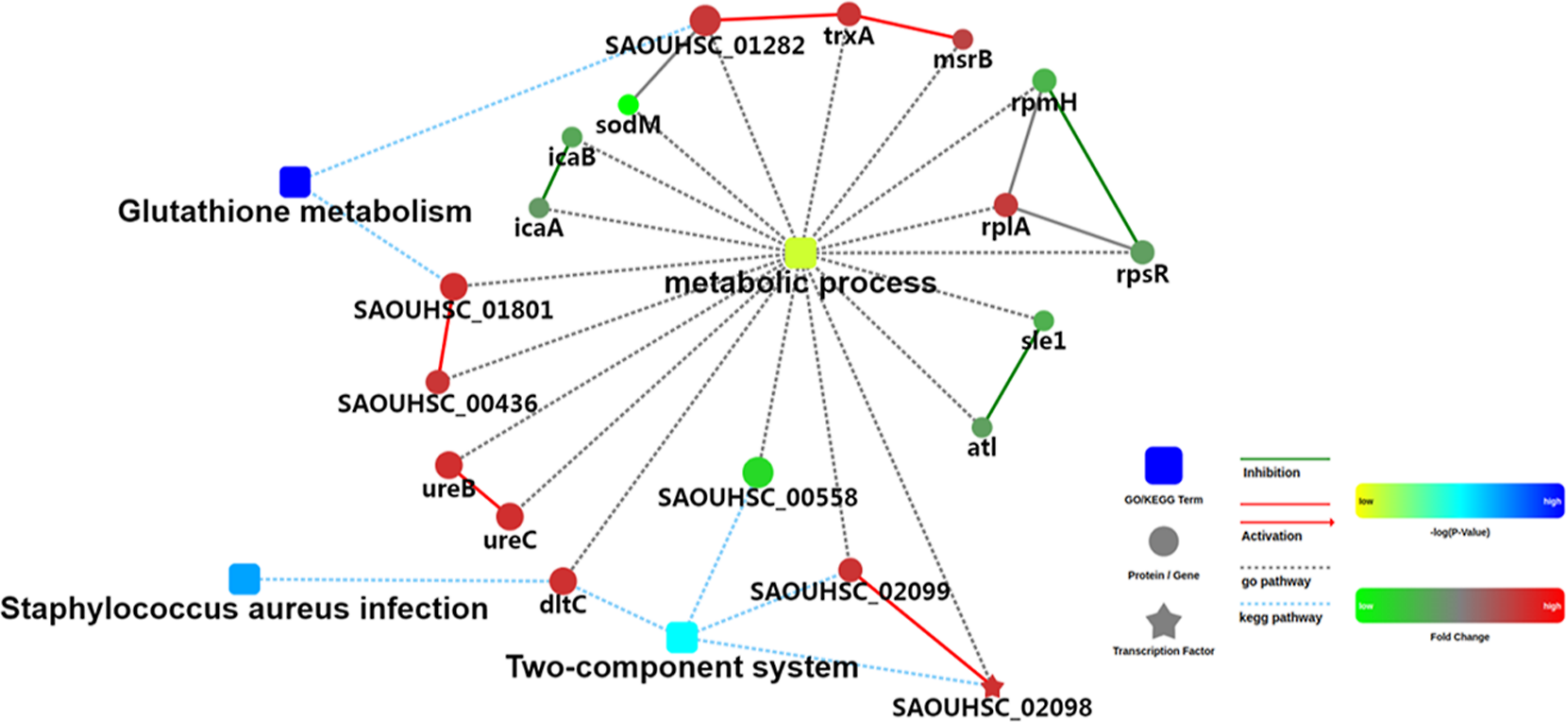
Protein–protein interaction network of different abundance proteins in the pinaverium bromide-treated *S. aureus* isolate. The protein–protein interaction network of different abundance proteins was analyzed through the STRING database.

In order to explore the mechanism of the inhibitory effect of pinaverium bromide on the growth, biofilm formation, and persisters of *S. aureus*, the different abundance proteins in the pinaverium bromide-treated *S. aureus* isolate were detected by proteome analysis in this study. There were 54 different abundance proteins determined (*P* ≤ 1.00 × 10^–2^, with fold-change ≥1.5 or ≤0.667) in the pinaverium bromide-treated *S. aureus* isolate and of which 33 proteins increased, whereas 21 proteins decreased (Figure 7 and Table S2). Based on GO annotation, the predominant biological process of different abundance proteins was involved in oxidative stress, detoxification, and metabolic process. The protein–protein interaction network of different abundance proteins in this study was analyzed through the STRING database (Figure 8). Interestingly, the abundance of superoxide dismutase (SOD) sodM significantly reduced. The abundance of ica locus proteins icaA and icaB also decreased. Meanwhile, the abundance of global transcriptional regulator spxA and Gamma-hemolysin component B (hlgB) increased in the pinaverium bromide-treated *S. aureus* isolate.

## Discussion

Pinaverium bromide, a gastrointestinal (GI)-selective calcium channel antagonist, inhibits the influx of calcium into intestinal smooth muscle cells, thus relieving the contraction of intestinal smooth muscles, defecation-associated abdominal pain, and discomfort in patients with irritable bowel syndrome (IBS).^14^ In recent years, pinaverium bromide was found to attenuate lipopolysaccharide (LPS)-induced excessive systemic inflammation via inhibiting neutrophil priming and protected the liver and lung from LPS-induced damage and reduced organ-specific inflammatory responses.^15^ However, there is no report on the antibacterial activity of pinaverium bromide, either for Gram-positive or Gram-negative bacteria. This study first found that pinaverium bromide had antibacterial activity against *S. aureus* and *E. faecalis*. Interestingly, the present research also demonstrated that pinaverium bromide could not only rapidly kill *S. aureus* and *E. faecalis* planktonic cells but also significantly inhibit the production of persister cells.

IBS, a chronic nonfatal illness, is commonly encountered in clinical practice; however, treatment options are limited and often ineffectual. In addition, there is increasing evidence that bacterial overgrowth in the bowel (dysbiosis) may be an etiological factor in IBS, and these overgrown pathogenic bacteria also include *S. aureus*.^16,17^ This means that in addition to the treatment of IBS by regulating calcium into intestinal smooth muscle cells, pinaverium bromide may also benefit patients by inhibiting the growth of intestinal *S. aureus*. In recent years, it was found that there were also mucosal biofilms formed by pathogenic bacteria on the intestinal mucosa of patients with IBS, which was an important reason for the disease to persist and recur.^18^ Although the role of *S. aureus* in the pathogenesis of mucosal biofilm in IBS has not been reported yet, this study first found that pinaverium bromide can inhibit the biofilm formation of *S. aureus*, which is expected to provide a new idea and direction for the treatment of mucosal biofilm infection in IBS.

The present research demonstrated that pinaverium bromide had a rapid bactericidal effect on *S. aureus* and *E. faecalis* planktonic cells, significantly reduced the production of persister cells, and was more effective than linezolid, vancomycin, and ampicillin. Ampicillin and vancomycin achieved antibacterial activity against Gram-positive bacteria by interfering with the cell wall synthesis, while linezolid obtained antibacterial activity by interfering with the protein synthesis of Gram-positive bacteria.^19^ Therefore, this may suggest that pinaverium bromide obtains antibacterial activity against *S. aureus* and *E. faecalis* not through interfering with the cell wall or protein synthesis. Another antispasmodic drug for IBS, otilonium bromide, has been found that had strong bactericidal activity against *S. aureus*. Otilonium bromide changed the permeability of the *S. aureus* membrane and caused membrane damage.^20^ The present study also found that the membrane polarity of *S. aureus* was significantly disrupted by pinaverium bromide. Interestingly, this study also found that the antibacterial activity of pinaverium bromide against *S. aureus* can be decreased by the over-supplemented cell membrane phospholipids phosphatidyl glycerol and cardiolipin. This means that pinaverium bromide, like otilonium bromide, also obtains antibacterial activity by damaging the cell membrane of *S. aureus*, and phosphatidyl glycerol and cardiolipin in the cell membrane may be its main target molecules.

In order to investigate the mechanism of pinaverium bromide against *S. aureus*, this study detected the different abundance proteins in the pinaverium bromide-treated *S. aureus* isolate by proteome analysis. Interestingly, this study found that the abundance of SOD sodM and ica locus proteins icaA and icaB decreased in the pinaverium bromide-treated *S. aureus* isolate. SOD sodA and sodM played an important role in the oxidative stress of *S. aureus*, and it was also found that SOD was related with biofilm formation of *Listeria monocytogenes* through coping with the oxidant burden in deficient antioxidant defenses.^21,22^ Therefore, pinaverium bromide may also inhibit the biofilm formation of *S. aureus* through sodM. Previous study reported that under oxidative stress, sodM became a major source of activity during the late exponential and stationary phases of growth of *S. aureus*.^23^ Under the pressure of antimicrobials, the stationary phase of growth is the key period for the formation of *S. aureus* persister cells.^24^ Thus, the formation of *S. aureus* persister cells was significantly inhibited by pinaverium bromide may also be through decreasing the function of sodM. The significant role of icaADBC-encoded polysaccharide intercellular adhesin (PIA) in staphylococcal biofilm formation and development has been well studied.^25,26^ The present study indicated that ica locus proteins icaA and icaB decreased in the pinaverium bromide-treated *S. aureus* isolate, this suggests that the inhibition of the expression of icaA and icaB, may be one of the important ways of the inhibition of the biofilm formation of *S. aureus* by pinaverium bromide.

## Conclusions

This study found that pinaverium bromide had an antibacterial effect on *S. aureus*. Moreover, the present research also demonstrated that pinaverium bromide could not only rapidly kill *S. aureus* planktonic cells but also significantly inhibit the production of *S. aureus* persister cells. Subinhibitory concentrations of pinaverium bromide significantly inhibited biofilm formation of *S. aureus*. Pinaverium bromide obtained antibacterial activity by damaging the cell membrane of *S. aureus*, and phosphatidyl glycerol and cardiolipin in the cell membrane may be its main target molecules. The SOD sodM and ica locus proteins icaA and icaB may be important approaches for the inhibitory effect of pinaverium bromide on the formation of biofilm and persisters of *S. aureus*.

## Methods

### Bacterial Isolates and Growth Conditions

A total of 54 *S. aureus* and 4 *E. faecalis* clinical isolates were used in this study, and these isolates were collected from Shenzhen Nanshan People’s Hospital (Grade A, level III Hospital, 1500 beds) between January 1, 2019, and December 31, 2021. All clinical isolates were identified with a Phoenix 100 automated microbiology system (BD, Franklin Lakes, NJ, USA) and were re-identified with matrix-assisted laser desorption ionization time-of-flight mass spectrometry (IVD MALDI Biotyper, Germany). The *S. aureus* ATCC29213, *S. aureus* SA113 (ATCC35556), and *E. faecalis* ATCC29212 were used as reference strains and were purchased from American Type Culture Collection (ATCC).

All the strains were grown in tryptic soy broth (TSB) at 37 °C with shaking of 180 rpm unless otherwise stated. For the antimicrobial susceptibility test and time-killing assay, strains were grown in a cation-adjusted Mueller–Hinton broth (CAMHB) at 37 °C with shaking. Strains were grown in TSBG (TSB with 0.5% glucose) at 37 °C for biofilm assay.

### Antimicrobials or Chemicals

Ampicillin sodium (catalogue no. HY-B0522A, purity: ≥98.0%), vancomycin (catalogue no. HY-B0671, purity: 98.11%), linezolid (catalogue no. HY-10394, purity: 99.95%), pinaverium bromide (catalogue no. HY-111613, purity: 99.83%), fluorescent dye DIBAC4(3) (catalogue no. HY-101892, purity: ≥98.0%), and chemicals screening libraries (ID: HY-LD-000001025, containing 1333 chemicals, now as part of the FDA-Approved Drug Library HY-L022) were purchased from MedChemExpress (MCE, Shanghai, China). Phosphatidyl choline (catalogue no. L130331, purity: >99.0%) and phosphatidyl glycerol (catalogue no. L130372, purity: >99.0%) were purchased from Aladdin (Aladdin, Shanghai, China). Cardiolipin (catalogue no. C0563, purity: ≥97.0%) and phosphatidyl ethanolamine (catalogue no. Y0001953, purity: European Pharmacopoeia Reference Standards) were purchased from Sigma-Aldrich (Sigma-Aldrich, Shanghai, China).

### Antimicrobial Susceptibility Testing

The MICs and MBCs of pinaverium bromide against *S. aureus* and *E. faecalis* isolates were determined by the broth macrodilution method in CAMHB according to the Clinical and Laboratory Standards Institute guidelines (CLSI-M100-S27). The MICs of ampicillin, vancomycin, and linezolid against *S. aureus* CHS101 and *E. faecalis* 16C106 isolates were also determined by the broth macrodilution method.

### Time-Killing Assay

The *S. aureus* CHS101 and *E. faecalis* 16C106 isolates in the logarithmic growth phase were used to explore the rapid bactericidal activity of pinaverium bromide and compared with linezolid, vancomycin, and ampicillin by the time-killing assay. The time-killing assay was conducted according to previous study.^12^ Briefly, overnight cultures were diluted 1:200 in fresh CAMHB and were cultured to the middle logarithmic growth phase (3.5 h), and then, pinaverium bromide, linezolid, vancomycin, and ampicillin were added (all at 4 × MIC) and were further incubated at 37 °C with shaking. At the time points of 2, 4, 6, and 24 h, the numbers of cfu were determined. All experiments were performed in triplicate.

The *S. aureus* CHS101 and *E. faecalis* 16C106 isolates in the stationary growth phase were used to explore the inhibitory effect of pinaverium bromide on the formation of persister cells and compared with linezolid, vancomycin and ampicillin by the time-killing assay. According to previous study,^12^*S. aureus* and *E. faecalis* isolates were cultivated overnight in CAMHB at 37 °C for 12 h to the stationary growth phase, and then, pinaverium bromide, linezolid, vancomycin, and ampicillin were added (all at 10 × MIC) and were further incubated at 37 °C with shaking. At the time points of 24, 48, 72, 96, and 120 h, the numbers of cfu were determined. All experiments were performed in triplicate.

### Biofilm Biomass Detected by Crystal Violet Staining

The biofilm biomass was detected by crystal violet staining, and chemicals with an inhibitory effect on the biofilm formation of *S. aureus* were screened from FDA-Approved Drugs Library according to our previous research.^11^ The biofilm formation of *S. aureus* was inhibited by pinaverium bromide: *S. aureus* was inoculated into 96 polystyrene microtiter plates with TSBG (with or without pinaverium bromide), and after 24 h of static incubation, the biofilms were detected by crystal violet staining. The effect of pinaverium bromide on the established biofilm of *S. aureus*: *S. aureus* was inoculated into 96 polystyrene microtiter plates with TSBG, and after static incubation for 24 h at 37 °C (mature biofilms established), the supernatants were removed and plates were washed, then pinaverium bromide was added, and were further static incubated for 24 h at 37 °C, and the remaining biofilms were detected by crystal violet staining. All the biofilm experiments were performed in triplicate at least three independent times.

### Detection of the Adherent Cells in Biofilms

The adherent cells in biofilms of the *S. aureus* CHS101 and *E. faecalis* 16C106 isolates were detected by the cfu numbers according to previous study.^11^ Overnight cultures of *S. aureus* and *E. faecalis* were 1:200 diluted with TSBG and inoculated into six polystyrene microtitre plates (with or without pinaverium bromide). After 24 h of static incubation at 37 °C, the supernatant was discarded and plates were washed thrice with 0.9% saline, the remaining adherent cells in biofilms were scraped using a cell scraper, and the numbers of cfu were determined. The experiment was performed in triplicate.

### Detection of Cell Membrane Polarity

The cell membrane polarities of the *S. aureus* CHS101 and *E. faecalis* 16C106 isolates were detected by fluorescent dye DIBAC4(3) staining according to previous study.^27^ DiBAC4(3) was a fluorescent dye and sensitive to membrane potential. Briefly, *S. aureus* CHS101 and *E. faecalis* 16C106 isolates’ suspension (OD_600_ = 0.5) was added into a black, opaque, flat-bottomed 96-well plate, followed by the incubation with the addition of DiBAC4(3) (1 μM) at 37 °C in the dark for 10 min. Subsequently, pinaverium bromide was added into each well with the final concentrations from 1/4 × MIC to 10 × MIC. The wells containing no pinaverium bromide was used as the negative control, and 0.1% TritonX-100 was used as the positive control. The fluorescence intensity was detected at 492 and 515 nm. Results were expressed in a relative fluorescence unit. All experiments were performed in triplicate.

### Detection of the Effect of Membrane Phospholipids in the Antibacterial Activities of Pinaverium Bromide

The effect of membrane phospholipids in the antibacterial activities of pinaverium bromide was determined by checkerboard microdilution methods based on previous study.^28^ Pinaverium bromide was diluted longitudinally with TSB, and membrane phospholipids were diluted transversely and inoculated in 96-well plates. Bacterial suspension was inoculated and was static incubated for 24 h at 37 °C. The fold changes of MICs of pinaverium bromide against *S. aureus* CHS101 and *E. faecalis* 16C106 isolates were determined.

### Cytotoxicity Assay

Human hepatocellular carcinoma cells HepG2 and Huh7, mouse monocyte-macrophage cell J774, and human hepatic stellate cell LX-2 were used to explore the cytotoxicity of pinaverium bromide by the Cell Counting Kit-8 (MCE) according to previous study.^29^ Cells were cultured at 37 °C in an atmosphere with 95% air and 5% CO2 in a Dulbecco’s Modified Eagle Medium (DMEM) containing 10% FBS, 100 U/mL penicillin, and 100 μg/mL streptomycin. Briefly, the cultured cells were seeded in a 96-well plate at 1.25×10^4^ cells/well. After cultured for 24 h, the cells were incubated with pinaverium bromide at different concentrations (100, 50, 25, 12.5, 6.25, 3.125, and 1.56 μM). Cells without pinaverium bromide were used as the control group. After 24 h of pinaverium bromide treatment, 10 μL of CCK8 solution/well was added and incubated at 37 °C for 1.5 h. The absorbance of each well at 450 nm (OD_450_) was measured. The cell viability (%) was calculated: OD_450_ of experimental group/OD_450_ of control group × 100%. All the cytotoxicity assays were performed in triplicate at least three independent times.

### Protein Extraction

In order to explore the mechanism of pinaverium bromide against *S. aureus*, the proteomics analysis was performed in *S. aureus* CHS101 with the treatment of pinaverium bromide (at 1/2 × MIC) for 4 h. Bacterial cultures was harvested at OD_600_ ∼0.8. The pellet was transferred to a precooled screw-cap tube, which was then added with 1.5 volumes of zirconia/silica beads (Biospec, 0.1 mm) and RIPA lysis buffer (Beyotime Biotechnology, China). The cells were lysed with a cell disruption device. The protein concentrations were measured by a commercial BCA assay.

### Proteomics Analysis

The harvested protein was reduced with 10 mM DTT (Sigma-Aldrich Co., St. Louis, MO) for 1 h at 70 °C, followed by alkylation using 50 mM iodoacetamide (IAA, Sigma-Aldrich) for 15 min at room temperature. The proteins were desalted and washed with ammonium bicarbonate by using Amicon Ultra Centrifugal Filters (10 kDa cutoff; Millipore, Billerica, MA) and were digested with trypsin (Promega, Madison, WI). LC–MS/MS analyses were performed with an UltiMate 3000 RSLC nanosystem coupled to a Q Exactive Plus mass spectrometer (Thermo Scientific). The protein identification and quantification were conducted using a Proteome Discoverer 2.4 base with the Sequest HT against the Uniprot reference proteome of *S. aureus* (strain NCTC 8325/PS47). The minimum unused score of 1.3 (equivalent to 95% confidence) and a false discovery rate less than 1% were required for all reported proteins. The different abundance proteins were uploaded into the OMICSBEAN database (http://www.omicsbean.com) for GO (gene ontology) annotation, including biological process, cellular component, molecular function, and KEGG pathway analysis and PPI networks.

### Statistical Analysis

All statistical analyses were performed with SPSS software (version 20.0, Chicago, IL, USA) using the Student’s *t*-test. Values of *P* < 0.05 were considered statistically significant.

## Supporting Information Available

MICs and MBCs of pinaverium bromide, global different abundances of proteins between the pinaverium bromide-treated *S. aureus* isolate and its control isolate, and effect of different concentrations of pinaverium bromide on the established biofilms (PDF)

## Notes

All procedures involving human participants were performed in accordance with the ethical standards of Shenzhen Nanshan People’s Hospital and the 6th Affiliated Hospital of Shenzhen University Medical School and with the 1964 Helsinki declaration and its later amendments, and this study was approved by the ethics committee of the Shenzhen Nanshan People’s Hospital and the 6th Affiliated Hospital of Shenzhen University Medical School. Isolates were collected as part of the routine clinical management of patients according to the national guidelines in China.
