# Supplementary material for: In Vitro Inhibition of Growth, Biofilm Formation, and Persisters of Staphylococcus aureus by Pinaverium Bromide
Source: ACS Omega. 2023 Mar 6;8(10):9652–61. doi: 10.1021/acsomega.3c00340 (PMC10018691; doi:10.1021/acsomega.3c00340)
Supplement: Supplementary file 1 — ao3c00340_si_001.pdf [file ao3c00340_si_001.pdf]

In vitro inhibiting growth, biofilm formation and persisters of *Staphylococcus aureus* by Pinaverium bromide

Ting Mao[1#], Bao Chai[2#], Yanpeng Xiong[3#], Hongyan Wang[3#], Lei Nie[4], Renhai Peng[3], Peiyu Li[3], Zhijian Yu[3], Fang Fang[4*], Xianqiong Gong[1*]

[1]Hepatology Center, Xiamen Hospital, Beijing University of Chinese Medicine, Xiamen 361001, China.

[2]Department of dermatology, Shenzhen Nanshan People's Hospital and the 6th Affiliated Hospital of Shenzhen University Medical School, Shenzhen 518052, China.

[3]Department of Infectious Diseases and Shenzhen Key Lab of Endogenous Infection, Shenzhen Nanshan People's Hospital and the 6th Affiliated Hospital of Shenzhen University Medical School, Shenzhen 518052, China.

[4]Department of Infectious Diseases and department of general medicine, the Key Lab of Endogenous Infection, Shenzhen Nanshan People's Hospital and the 6th Affiliated Hospital of Shenzhen University Medical School, Shenzhen 518052, China.

[#] Ting Mao, Bao Chai, Yanpeng Xiong and Hongyan Wang contributed equally to this work.

[*]**Corresponding author**:

Fang Fang, 710194599@qq.com;

Xianqiong Gong, xianqiong-gong@hotmail.com;

Table S1. The MICs and MBCs of pinaverium bromide against *S. aureus* and *E. faecalis*.

Bacterial species	Isolates	Antimicrobial susceptibilities of pinaverium bromide	
		MIC (μM)	MBC (μM)
**MSSA**			
	ATCC29213	12.5	50
	YUSA10	12.5	25
	YUSA61	25	50
	YUSA75	12.5	50
	YUSA77	25	50
	YUSA78	12.5	50
	YUSA80	12.5	50
	YUSA81	12.5	50
	YUSA82	12.5	50
	YUSA83	12.5	50
	YUSA87	12.5	25
	YUSA90	12.5	25
	YUSA92	12.5	50
	YUSA94	12.5	50
	YUSA103	6.25	25
	YUSA106	25	100
	YUSA107	25	50
	YUSA108	12.5	50
	YUSA110	12.5	50
	YUSA126	12.5	100
	YUSA128	12.5	25
	YUSA134	25	50
	YUSA135	12.5	100
	YUSA140	12.5	50
	YUSA152	12.5	12.5
	CHS46	12.5	50
	CHS101	25	25
	CHS128	12.5	50
	SA113	12.5	50
	YUSA73	12.5	50
	YUSA74	12.5	25
	YUSA76	12.5	50
	YUSA85	12.5	100

	YUSA97	12.5	100
	YUSA105	12.5	100
	YUSA117	12.5	50
	YUSA139	50	100
	YUSA144	50	100
	YUSA145	50	100
	YUSA213	12.5	100
**MRSA**			
	HaMRSA129	100	100
	YUSA142	12.5	25
	YUSA218	12.5	100
	CHS350	12.5	50
	CHS563	12.5	25
	CHS655	12.5	25
	CHS670	12.5	25
	CHS684	12.5	100
	CHS686	12.5	50
	CHS692	12.5	100
	CHS707	12.5	50
	CHS712	25	100
	CHS723	50	100
	CHS727	12.5	25
	CHS767	12.5	25
	CHS779	12.5	50
E. faecalis			
	ATCC29212	25	100
	16C51	50	>100
	16C106	50	100
	16C166	50	>100
	16C350	25	50

Note: MIC, minimum inhibitory concentration; MBC, minimum bactericidal concentration; MSSA, methicillin-sensitive *S. aureus*; MRSA, methicillin-resistant *S. aureus*;

Table S2. Global different abundance of proteins between the pinaverium bromide treated *S. aureus* isolate and its control isolate.

Uniprot ID	Gene name	Proteins	Fold change	*P*-value
**Abundance decreased**				
Q2G261	sodM	Superoxide dismutase [Mn/Fe] 2	0.1265	1.08E-02
Q2G0K2	SAOUHSC_00558	Acetyl-CoA acetyltransferase, putative	0.2357	3.09E-02
Q2FUQ0	rpmH	50S ribosomal protein L34	0.4308	5.54E-03
Q2FVS7	SAOUHSC_02614	Aldose 1-epimerase	0.4404	4.90E-02
Q2FVR0	hrtB	Putative hemin transport system permease protein HrtB	0.4404	3.18E-02
Q2G0U9	sle1	N-acetylmuramoyl-L-alanine amidase sle1	0.4601	1.01E-03
Q2FZH7	SAOUHSC_01027	DUF697 domain-containing protein	0.4623	3.36E-05
Q2FZC2	SAOUHSC_01110	Fibrinogen-binding protein	0.4750	1.25E-03
Q9RQP7	icaB	Poly-beta-1,6-N-acetyl-D-glucosamine N-deacetylase	0.5267	8.91E-03
Q2FXT3	ruvA	Holliday junction ATP-dependent DNA helicase RuvA	0.5337	2.94E-03
Q2G0Z4	SAOUHSC_00367	L-cystine transporter	0.5598	4.17E-05
Q2FXZ8	SAOUHSC_01677	NfeD domain-containing protein	0.5703	4.57E-02
Q2FZK7	atl	Bifunctional autolysin	0.5987	3.49E-03
Q2G111	rpsR	30S ribosomal protein S18	0.6075	2.78E-03
Q2G1V0	SAOUHSC_02687	Formate/nitrite transporter, putative	0.6177	1.43E-02
Q2FZ71	pyrF	Orotidine 5'-phosphate decarboxylase	0.6229	6.91E-04
Q2FVK5	sbi	Immunoglobulin-binding protein Sbi	0.6242	5.92E-03
Q2FVS4	SAOUHSC_02618	ABC transporter permease	0.6289	8.91E-04
Q2G2P8	nnrD	ADP-dependent (S)-NAD(P)H-hydrate dehydratase	0.6502	2.72E-02
Q9RQP9	icaA	Poly-beta-1,6-N-acetyl-D-glucosamine synthase	0.6598	9.40E-05
Q2G0W8	SAOUHSC_00406	Lipase_3 domain-containing protein	0.6639	1.56E-02
**Abundance increased**				
Q2G1C4	tarJ'	Ribulose-5-phosphate reductase 2	1.5011	4.46E-02
Q2G1U6	spxA	Global transcriptional regulator Spx	1.5094	1.00E-02
P0A088	msrB	Peptide methionine sulfoxide reductase MsrB	1.5104	8.77E-04
Q2G0E7	SAOUHSC_00659	TPR_REGION domain-containing protein	1.5126	6.16E-03
Q2G2L1	tagH	Teichoic acids export ATP-binding protein TagH	1.5316	1.14E-03
Q2FYN2	cspA	Cold shock protein CspA	1.5433	6.36E-03
Q2FYK2	SAOUHSC_01438	Nfu_N domain-containing protein	1.5454	8.31E-04
Q2FXI6	SAOUHSC_01860	Thioredoxin domain-containing protein	1.5757	9.67E-05
Q2G2S6	prsA	Foldase protein PrsA	1.5943	2.47E-04
Q2G0P0	rplA	50S ribosomal protein L1	1.5943	4.49E-02
Q2FYZ0	SAOUHSC_01282	Glutathione peroxidase	1.6290	3.05E-02
Q2FZH6	SAOUHSC_01028	Phosphocarrier protein HPr	1.6312	1.84E-02
Q2FZM5	SAOUHSC_00975	DoxX family protein	1.6403	1.04E-04
Q2G0U1	SAOUHSC_00436	NADH-glutamate synthase small subunit, putative	1.6552	2.09E-02
Q2FZD2	trxA	Thioredoxin	1.6598	3.70E-04
Q2FXY2	SAOUHSC_01698	CRM domain-containing protein	1.6609	1.72E-02
Q2G000	SAOUHSC_00834	Thioredoxin, putative	1.6632	1.45E-04
Q2FUQ5	SAOUHSC_03049	ParB domain-containing protein	1.6656	2.59E-04

Q2FX08	SAOUHSC_02099	Sensor protein VraS	1.6725	1.11E-02
Q2FX09	SAOUHSC_02098	Response regulator protein VraR	1.7041	2.01E-04
Q2FY06	era	GTPase Era	1.7053	9.00E-04
Q2FZW4	dltC	D-alanyl carrier protein	1.7065	1.01E-03
Q2FWD9	tdk	Thymidine kinase	1.7231	6.85E-03
Q2FUQ9	SAOUHSC_03045	Cold shock protein	1.7267	4.42E-03
Q2G2K5	ureC	Urease subunit alpha	1.7302	4.51E-03
Q2FXN4	SAOUHSC_01801	Isocitrate dehydrogenase [NADP]	1.7302	1.99E-02
Q2G2K6	ureB	Urease subunit beta	1.7339	1.58E-03
Q2G009	SAOUHSC_00819	Cold shock protein CspA	1.7605	1.03E-02
Q2FVG8	SAOUHSC_02744	Amino acid ABC transporter, ATP-binding protein, putative	1.7801	1.38E-02
Q2FX00	SAOUHSC_02106	Lipid II isoglutaminyl synthase (glutamine-hydrolyzing) subunit GatD	1.8570	1.44E-04
Q2FVK1	hlgB	Gamma-hemolysin component B	2.3086	7.40E-04
Q2FV69	SAOUHSC_02867	HTH tetR-type domain-containing protein	2.3246	1.20E-05
Q2FXV3	SAOUHSC_01728	Bac_luciferase domain-containing protein	2.3784	3.09E-03

The data are given as the means of the results from two independent experiment.

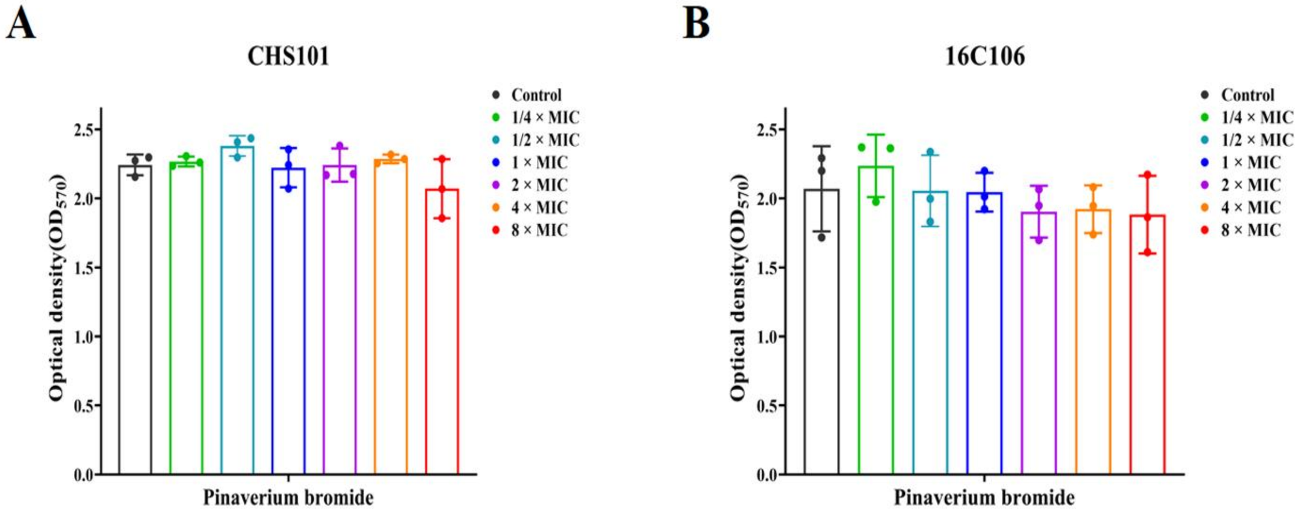

Figure S1 | The effect of different concentrations of pinaverium bromide on the established biofilms of *S. aureus* and *E. faecalis*. The *S. aureus* CHS101 isolate (**A**) and *E. faecalis* 16C106 isolate (**B**) formed mature biofilms for 24 h, then treated with pinaverium bromide at different concentrations for 24 h, and the remaining biofilm biomasses were determined by crystal violet staining. The data presented was the average of three independent experiments (mean ± SD). MIC, minimum inhibitory concentration;
